# Obituary

**Published:** 1888-04

**Authors:** 


### OBITUARY.

The late Prof. William Robertson was born at Dalkeith in Scotland in 1832, and was educated at Selkirk Grammar School, obtained the diploma of the Highland Agricultural Society in 1852, and became a member of the Royal College of Veterinary Surgeons in 1860 and a Fellow in 1877. He was for many years a member of the Council of the R. C. V. S., and an Examiner in Comparative Anatomy and Pathology. After graduating as a V. S., he practiced his profession at Kelso, where he earned the confidence and esteem of the inhabitants and was the recipient of several marks of respect. In 1867 he became the author of "Hints on Stockbreeding," which had a ready and large circulation; he was a frequent contributor to current veterinary medical literature, was chosen Principal of the Royal Veterinary College in 1881, and also became Chief Veterinary Adviser to the Royal Agricultural Society. In his capacity of director of the school he was untiring in his zeal, taking interest alike in the scientific methods of investigation and in the every-day clinical working of the establishment. As a teacher he was ever ready to impart that information which, by long experience and close observation he had gained during a most active life, wholly given up to the study of animal diseases. On the morning of his decease he had lectured as usual, but complained of hoarseness and palpitation of the heart. At the time of his death, which took place about 2:30 P. M. on the 15th of December, he was at work in his office, revising proofs of his work about to be published on "Veterinary Surgery." The companion volume to his "Equine Medicine," published in 1883.

The deceased gentleman was very popular with the students of the college, and was held in high esteem by his colleagues and numerous professional friends, who have determined to erect a bust to his memory, which, with the permission of the Governors, will be placed in the museum of the school. C. A. S.

